# Therapists’ experiences with non-verbal communication in videopsychotherapy: a qualitative interview study

**DOI:** 10.3389/fpsyg.2026.1785382

**Published:** 2026-08-24

**Authors:** Jolina Holzhaus, Christian Meyer-Keirath, Kristina Geue, Florian Junne, Hannah Wallis

**Affiliations:** 1 Medical Faculty, University Clinic for Psychosomatic Medicine and Psychotherapy, University Medicine, Otto Von Guericke-University Magdeburg, Magdeburg, Germany; 2 German Center for Mental Health (DZPG), Partner Site Halle-Jena-Magdeburg, Magdeburg, Germany; 3 Center for Behavioral Brain Sciences (CBBS), Magdeburg, Germany

**Keywords:** non-verbal communication, online therapy, telepsychotherapy, video-based psychotherapy, videotherapy, videopsychotherapy

## Abstract

**Background:**

Videopsychotherapy (VPT) has gained in importance since the Corona pandemic. As non-verbal communication (NVC) differs from the face-to-face (FtF) therapy, its practical assessment is crucial for long-term quality improvement.

**Objective:**

The aim of the study is to investigate how psychotherapists perceive specific aspects of NVC in VPT, in order to derive practical insights and lay the groundwork for future research approaches to improve treatment quality.

**Methods:**

Semi-structured expert interviews were conducted with twelve psychotherapists in Germany. The interviews took place from October 2024 to March 2025 and were analyzed using qualitative content analysis according to Mayring.

**Results:**

Certain aspects of NVC, such as facial expression, gestures, eye contact, body posture and self-view, were perceived as limited or altered in VPT, which increased the risk of misinterpretation. Having a self-view led to more control over one’s own non-verbal behavior. Therapists reported adapting their behavior by smiling and nodding more frequently, as well as making more eye contact with the camera.

**Conclusion:**

The study shows that NVC in VPT is experienced as limited, but can also offer potential. To ensure long-term improvement in treatment quality, further practical studies and development of specific interventions to improve NVC in video settings are required.

## 1 Introduction

In recent years, videopsychotherapy (VPT) has become significantly more important, primarily due to the COVID-19 pandemic (Steubl and Baumeister, 2023). A key difference between face-to-face (FtF) settings and video settings is the change in non-verbal communication (NVC; Leukhardt et al., 2021). For instance, direct eye contact is only possible to a limited extent due to technical constraints, and the view is typically restricted to the upper body. While NVC is acknowledged as an essential part of the therapeutic process, its specific role in the context of VPT remains largely unexplored (Smith et al., 2022).

The term “videotherapy” encompasses various activities, including counseling and treatments carried out by various healthcare professionals using digital communication tools (Smith et al., 2022). These therapeutic approaches belong to the field of telemedical care, enabling location-independent contact between providers and patients. This paper focuses on “videopsychotherapy” (VPT) as a specific form of videotherapy, in which psychotherapeutic treatment is conducted in real time via a video connection between therapist and patient.

The COVID-19 pandemic led to a substantial global increase in the use of videotherapy. Before the pandemic, only 7.1% of therapy sessions in the United States were conducted online. During lockdowns, this proportion rose to 85.5% (Pierce et al., 2021). Post-pandemic, videotherapy is expected to be used approximately five times more frequently than before the pandemic (Pierce et al., 2021). The study was conducted in Germany, where an increase in the use of videotherapy was also observed. The proportion of therapists offering videotherapy increased from 4.5% in 2017 to around 25% in 2021. Although usage temporarily declined thereafter, it rose again in 2024 and remains well above pre-pandemic levels (Die Techniker, 2025). In Germany, psychotherapeutic treatments are typically reimbursed through statutory health insurance. Specific regulations apply, stipulating that only a limited share of treatment cases may be conducted entirely via video. This limit, however, was expanded following the COVID-19 pandemic (BPtK, 2025). Alternatively, other reimbursement pathways exist, enabling some practices to provide therapy sessions entirely online to all of their patients (Pkv, 2023).

The regulatory and practical conditions appear to be evolving in Germany and internationally in a way that supports the implementation of VPT as a standard care option. In this context, research on videopsychotherapy and the preservation of treatment quality is becoming increasingly important (Smith et al., 2022). One aspect that may significantly contribute to treatment quality, which has been highlighted in current research as a key difference from FtF therapy, is NVC (Duffy and Benotsch, 2025). So far NVC processes are largely neglected in the existing guidelines for VPT (Church et al., 2023; Joint Task Force for the Development of Telepsychology Guidelines for Psychologists, 2013; Turvey et al., 2013). Recommendations primarily address technical aspects of visual and auditory signal quality, but do not formulate specific suggestions for the therapeutic handling of the changed NVC. A rapid review of 40 internationally published guidelines on VPT, also supports the perspective that NVC has not yet been treated as a central aspect in existing recommendations (Ekeleme et al., 2024).

Non-verbal communication encompasses all forms of interpersonal communication that do not involve spoken language. This includes posture, facial expressions, eye contact, gestures, spatial distance and touch, for example (Matsumoto et al., 2016). In a therapeutic context, NVC is crucial for establishing trust, managing interpersonal distance, offering emotional reflection, and recognizing empathy (Henry et al., 2012; Knapp et al., 2013).

In the context of VPT, initial research suggests that various technical factors may influence the perception of NVC. For example, early studies indicate that the quality of NVC perception can be affected by camera position, framing, transmission delays, and technical equipment (Duffy and Benotsch, 2025; Frittgen and Haltaufderheide, 2022). While basic emotional signals appear to remain identifiable, more subtle body language cues and nuanced emotional responses may be more difficult to interpret in VPT (Duffy and Benotsch, 2025). Surveys suggest, that limited NVC is frequently perceived as a disadvantage of VPT (Békés and Aafjes-van Doorn, 2020; Leukhardt et al., 2021). At the same time, preliminary findings indicate that some therapists develop strategies to better perceive or compensate for reduced non-verbal cues over time, such as offering more explicit verbal feedback, optimizing their technical setup, or adapting the therapeutic setting (García et al., 2022; Simpson and Reid, 2014). Furthermore, one study showed that therapists with higher non-verbal expressiveness perceived their VPT capabilities as less limited than therapists with lower non-verbal expressiveness (Lin and Anderson, 2024).

Previous research has demonstrated that important aspects of NVC are altered in VPT (García et al., 2022). However, most of this work have addressed NVC only in general terms and have treated it as a secondary rather than a primary focus of investigation (Duffy and Benotsch, 2025). To our knowledge, there is a lack of research examining specific dimensions of NVC in VPT in depth, including both the positive and negative consequences of altered NVC, as well as the adaptive communication strategies psychotherapists develop in response (Duffy and Benotsch, 2025).

This study extends previous research by explicitly focusing on selected aspects of NVC as a primary focus of investigation, such as facial expressions, gestures, eye contact, body posture, and self-view. Furthermore, it explores how therapists perceive changes in these specific aspects of NVC and how they adapt their communication and therapeutic practice in response. By providing a detailed account of therapists’ experiences, this study aims to deepen our understanding of the role of NVC in VPT and to lay the groundwork for future research and intervention studies in this field.

## 2 Material and methods

### 2.1 Overview

In this study, qualitative, semi-structured expert interviews were conducted to gain in-depth insights into experiences with specific aspects of NVC in VPT.

### 2.2 Participants

#### 2.2.1 Inclusion criteria

Specific inclusion criteria were defined to ensure that participants had sufficient experience in both FtF psychotherapeutic practice and VPT. The criteria were as follows:

1. At least 1 year of professional experience in the field of psychotherapy
2. Ongoing or completed licensure training in psychotherapy
3. Online individual sessions conducted with at least three different patients
4. A minimum of five video individual therapy sessions conducted

#### 2.2.2 Sampling and recruitment

Recruitment of participants took place between October 2024 and April 2025, partly in collaboration with a Master’s student in Psychology who also conducted interviews as part of her thesis. Psychotherapists were contacted through several channels. Staff members of the University Clinic for Psychosomatic Medicine and Psychotherapy in Magdeburg were invited to participate, as were additional therapists offering VPT across Germany (identified via platforms such as psychotherapie.de). Furthermore, invitations to participate in the study were shared among colleagues through snowball sampling.

In total, more than 200 psychotherapists were contacted by email. Most either did not respond or declined to participate, primarily due to time constraints or limited experience with videopsychotherapy. Approximately 10% expressed general interest in participating. However, interviews could not be conducted with several participants due to scheduling difficulties, lack of response or unmet inclusion criteria. Ultimately, 12 interviews were completed with psychotherapists who met the study’s inclusion criteria.

### 2.3 Ethical consideration

All procedures involving human participants were approved by the Ethics Committee of the Medical Faculty at the University Hospital of Magdeburg (approval number 134/24). Ethical approval was granted on 19 September 2024. All participants received a written information sheet detailing the study procedures and applicable data protection regulations. Prior to the interviews, participants were informed about the audio recording and provided written informed consent for anonymized processing and publication of the collected data. Participation was voluntary and no financial or material compensation was provided.

### 2.4 Interview procedures

#### 2.4.1 Development of the interview guide

A semi-structured interview guide was developed based on Helfferich’s framework (Helfferich, 2011). The guide was first discussed and revised within the research team. It then underwent an external review by a psychologist with extensive experience in qualitative research. Their methodological and content-related suggestions were incorporated into the guide. The guide was then tested in a trial interview with an experienced psychologist who had not been involved in its development. Initially, the interview guide included general questions about attitudes toward and use of VPT. It then addressed more specific topics, such as the particularities of NVC (e.g., eye contact, gestures, and facial expressions), therapists’ perceptions of their own NVC, and their perceptions of patients’ NVC in VPT. Therapists were also asked about the advantages and disadvantages of videotherapy with respect to NVC processes. Given the broad range of specific non-verbal aspects involved in therapeutic interactions, we chose to focus on selected aspects of NVC that we expected therapists to be able to describe in detail based on their clinical experiences. At the same time, the interview guide was intentionally semi-structured to provide open space for therapists to introduce further aspects of NVC that they perceived as relevant. The semi-structured interview guide is provided in Supplementary Material 1.

#### 2.4.2 Conducting the interviews

Data collection took place between December 2024 and April 2025. After participants received the information sheet and provided written consent regarding data protection, interview appointments were arranged via email. At the beginning of each interview, the procedure and the consent for audio recording and transcription were reviewed and verbally confirmed. All interviews were conducted individually via the video platform Zoom. The duration of the interviews ranged from 16 to 59 min (mean: 29 min). After each interview, the interviewer completed a protocol sheet documenting the interview duration, the type of initial contact, the overall interview atmosphere, and any notable occurrences during the conversation.

#### 2.4.3 Transcription and data management

The audio recordings were transcribed using AI-assisted transcription software that complies with data protection regulations. The resulting transcripts were then manually reviewed, anonymized and prepared for analysis by a native German speaker. Transcription followed the guidelines of Dresing and Pehl (2018). To improve readability, quotations were linguistically smoothed without altering their meaning, and omissions are indicated by […].

#### 2.4.4 Data analysis

Data analysis was conducted using MAXQDA and followed the principles of qualitative content analysis according to Mayring and Fenzl (2019). First, deductive categories were developed based on existing literature and the interview guide. These included predefined NVC categories eye contact, facial expressions, gestures, and self-view, which were discussed and refined within the research team before coding. During analysis, text segments were assigned to these categories whenever appropriate. If a text segment addressing multiple aspects of NVC, it could be assigned to the two most relevant categories to preserve the context and comprehensibility of participants’ statements. During the thematic analysis, however, each text segment was interpreted only with regard to the aspect relevant to the respective category. This ensured that overlapping coding enhanced interpretability without resulting in duplicate analyses or overrepresentation of individual statements. The interviews were coded sequentially, with inductive categories added for emerging themes and refined through team discussions as required. When new inductive categories emerged, previously coded interviews were revisited to identify text segments that fitted the newly developed categories, thereby ensuring the consistent application of the coding framework across the entire dataset. For example, body posture and verbalization emerged as recurring concepts that could not be adequately captured by the initial coding framework and were therefore incorporated as additional categories. As the analysis progressed, no new relevant categories emerged, indicating thematic saturation. Finally, the coded segments were organized thematically and summarized in tables to present key statements and recurring patterns systematically. The Code System is provided in Supplementary Material 2.

### 2.5 Quality assurance

The quality criteria of this study were guided by the COREQ standard (Consolidated Criteria for Reporting Qualitative Research) (Tong et al., 2007). The COREQ-Checklist is provided in Supplementary Material 3.

#### 2.5.1 Reliability testing

To evaluate the consistency of coding decisions over time, four interviews were recorded by the same researcher after a 4-week interval (Rössler, 2017). Agreement rates ranged from 80.65% to 96.77%, with an average Cohen’s kappa of 0.90. According to the classification by Landis and Koch, this represents almost perfect agreement, the highest category of agreement, indicating a high level of consistency between coding decisions across different time points (Landis and Koch, 1977). To assess the consistency and comprehensibility of the coding framework, one transcript was randomly selected and independently coded by an experienced master’s student in psychology. Discrepancies were subsequently discussed within the research team and did not require substantial revisions of the coding framework. The resulting kappa value of 0.65 indicates substantial agreement according to the classification by Landis and Koch, reflecting strong consistency between raters (Landis and Koch, 1977).

### 2.6 Positionality statement

The study was conducted as a subproject of the research initiative “PsyPan” within the working group “Psychische Gesundheit im Kontext und Wandel” (Mental Health in Context and Change) at the University Clinic of Psychosomatic Medicine and Psychotherapy, University Hospital Magdeburg. The research team involved in the interview study included individuals with psychotherapeutic practice experience, academic researchers, and medical and psychology professionals. Their expertise in qualitative research ranged from basic knowledge to advanced methodological proficiency. The interviews were conducted by a German-speaking medical student trained in interviewing techniques and qualitative methodology. The interviewer had no prior practical experience with VPT outside of medical training, which may have facilitated an open and less assumption-driven interviewing style. At the same time, therapists may have adapted their explanations by providing more background information than they would have to an experienced psychotherapist. The development of the interview guide, data collection, and analysis were carried out in close and continuous collaboration with the research team.

## 3 Results

### 3.1 Overview

At the time of the interviews, participants had an average age of 39.5 years. In addition to the information presented in the Table 1, further contextual data were collected, including professional background: nine participants were psychological psychotherapists and three were medical psychotherapists. Where applicable, their licensure completion years ranged from 1999 to 2023. Most worked in private outpatient practice, with a smaller number employed in inpatient or outpatient clinical settings. Three therapists conducted psychotherapy exclusively online, while the majority conducted most of their sessions FtF and only a smaller proportion online. Most interviewed therapists worked in their own private practices. The sample included therapists from different theoretical orientations, with cognitive behavioral therapy (CBT) being the predominant approach.

**TABLE 1:** Participant characteristics.

Interview	Gender	Age	Training	Psychotherapeutic experience (years)	Online video sessions**	Proportion of videotherapy
1	F	29	in tr. CBT*	>5 yrs	>10	<10%
2	F	35	CBT*	>10 yrs	>50	>10%
3	F	40	PDT*	>20 yrs	>50	>20%***
4	F	38	CBT*	>20 yrs	>50	>20%***
5	F	61	PDT*	>20 yrs	>50	>10%
6	F	35	CBT*	>10 yrs	>50	>10%
7	F	35	CBT*	>5 yrs	>40	>10%
8	F	38	CBT*	>10 yrs	>50	>20%***
9	F	38	CBT*	>10 yrs	>40	<10%
10	F	37	CBT*	>10 yrs	>40	<10%
11	M	34	PDT*	<10 yrs	>30	<10%
12	M	54	PDT*	>10 yrs	>50	<10%

*CBT, cognitive behavioral therapy; in tr. CBT, in training for CBT licensure; PDT, psychodynamic psychotherapy. Psychoanalysis is officially recognized as a distinct guideline-based modality in Germany but was categorized together with psychodynamic psychotherapy for the purposes of analysis. **Estimated number of sessions reported by participants. ***Private practice only; at the time of the interviews, therapy was conducted exclusively online.

### 3.2 Main results

The following section presents the key thematic aspects of each category. The inserted subheadings are provided for clarity and do not represent independently developed categories. Selected quotes have been linguistically smoothed and shortened for readability, without altering their original meaning.

#### 3.2.1 Facial expression

##### 3.2.1.1 Facial expressions perceived as comparable but used more consciously

Five of the interviewed therapists reported that their patients’ facial expressions were just as perceptible in VPT sessions as in FtF settings, provided that the technical quality and lighting conditions were sufficient. Two therapists noted that they intentionally smiled more or nodded more frequently during video sessions.

*“I do it more noticeably, especially the nodding. And I actually find that really helpful […] because you have a relatively small area to focus on.” (Interview 2)*

##### 3.2.1.2 Subtle facial cues are perceived as less visible.

Five therapists reported difficulty perceiving subtle facial expressions and micro-signals, such as blushing, sweating or stress reactions. This was particularly the case when they knew their patients only from video consultations and when the camera quality was poor. It was also noted that tears were sometimes recognized with a delay.

*“What I probably wouldn’t be able to notice is if the person turns red or starts sweating […]. That’s harder to recognize, depending on the camera quality.” (Interview 1)*

##### 3.2.1.3 The strong focus on the face may lead to over-intimacy

Two therapists pointed out that the close proximity to the camera may create a sense of over-intimacy. This can cause discomfort or misunderstandings in emotionally unstable or structurally vulnerable patients, for example, when facial expressions such as frowning are interpreted as signs of disapproval. One therapist described that, due to the strong focus on the face, particularly intense facial expressions in the video setting can feel more difficult to tolerate.

*“She often had such a pained facial expression, which I perceived even more intensely in that setting […]. It was also a major challenge for me to endure that.” (Interview 11)*

##### 3.2.1.4 The strong focus on the face can sometimes make analysis easier

Three therapists reported that they were sometimes able to analyze their patients’ facial expressions more effectively in the video setting than in FtF sessions, as the face was more prominently in focus. This enabled more targeted observation of subtle facial reactions, eye movements and gaze direction. This was perceived as partially compensating for the loss of other non-verbal cues, such as gestures.

*“You can read much more from the face […]. All the concentration is directed there.” (Interview 11)*

##### 3.2.1.5 Facial expression results summary

Comparable to FtF therapy: Five of the interviewed psychotherapists perceived patients’ facial expressions during VPT to be comparable to those observed in FtF therapy.

Challenges: Five participants reported that facial expressions were more difficult to interpret in the video setting, particularly subtle facial cues and micro-expressions, such as blushing, sweating, tears or stress reactions. In addition, two psychotherapists described an increased sense of intimacy resulting from the close-up view of the patient’s face on the screen.

Perceived advantages: Three psychotherapists considered the close-up view of the face to be beneficial, as it enabled a more detailed observation and interpretation of facial expressions.

Adaptation strategies: Two psychotherapists reported that they deliberately smiled more frequently during VPT.

#### 3.2.2 Gestures

##### 3.2.2.1 Loss of information due to limited visibility of gestures

All psychotherapists described gesture as being generally less visible in the VPT, with ten participants explicitly identifying this limitation as a relevant loss of non-verbal information. Particularly gestures involving the lower body such as leg movements or fidgeting with the hands under the table as a means of releasing tension, were hardly perceptible in the video setting. This made it more difficult to assess emotions such as fear, nervousness or tension.

*“I was sure that one patient was playing with her hands, but only because I’ve known her for a long time. Otherwise, I would have missed it. It’s clearly a loss of information.” (Interview 11)*

*“What I sometimes find really insufficient is this small section. I can’t see what the hands or feet are doing, even though they actually communicate a lot” (Interview 5)*

##### 3.2.2.2 Restriction of gesticulation due to external factors:

Three therapists reported that external conditions in videotherapy sessions could further restrict gesticulation. For example, patients may need to use their hands to adjust the camera position. One therapist also noted that patients in video therapy sessions tended to engage in distracting activities more frequently, such as repeatedly drinking or looking at their phones.

*“As patients often hold the laptop, it is difficult to make gestures.” (Interview 10)*

##### 3.2.2.3 Adaptation strategies to enhance gesture visibility

Two psychotherapists reported that they consciously or unconsciously adapted their gestures during VPT. To convey non-verbal signals despite the restricted field of view, they raised their hands more frequently or positioned them closer to their face to increase the visibility of their gestures.

*“When I explain a model and need to use my hands, I make sure to raise them so that they are visible to the camera […] I also check whether my hands are within the camera frame.” (Interview 8)*

##### 3.2.2.4 Increase in gestures within the patients’ facial area

One psychotherapist described an increase in gestural interactions within the patients’ facial area, which may provide additional non-verbal information compared to FtF therapy.

*“Patients often have their hands near their face, which sometimes even results in more non-verbal interaction than in the practice setting, where their hands are resting on their lap.” (Interview 6)*

##### 3.2.2.5 Gestures results summary

Comparable to FtF therapy: Two psychotherapists reported that, despite reduced visibility of gestures, they did not perceive a relevant loss of information.

Challenges: Ten psychotherapists reported a relevant loss of information due to reduced visibility of gestures. This made it more difficult to identify signs of tension or nervousness accurately. Additionally, three psychotherapists described external factors, such as holding a smartphone during the session, as further restricting gestural communication.

Perceived advantages: One psychotherapist described an increase in gestural interactions within the visible camera frame, which may provide additional information compared to FtF therapy.

Adaptation strategies: Two psychotherapists reported intentionally moving their hands more frequently within the visible camera frame to enhance the perception of gestures.

#### 3.2.3 Eye contact and gaze behavior

##### 3.2.3.1 Eye contact is perceived as important but challenging

Ten therapists described direct eye contact as an important aspect of VPT. Two therapists reported that they did not perceive any relevant loss of non-verbal information compared with FtF sessions, as long as camera positioning and video quality were adequate. However, eight therapists found establishing and perceiving eye contact in videotherapy technically and situationally challenging. It was emphasized in particular that direct eye contact is affected by technical conditions: in order to create the impression of direct eye contact while looking at the conversation partner on the screen, it is necessary to look into the camera. This was described as potentially irritating or unnatural. One therapist reported actively trying to look into the camera to convey a sense of eye contact to the patient.

*“When the patient looks at my image, it doesn’t look like they are looking at me. That can be unsettling.” (Interview 11)*

*“A silent agreement is missing. Protest or setting boundaries is easier to notice. But this positive connection through eye contact is somewhat disrupted.” (Interview 12)*

##### 3.2.3.2 Diagnostic limitations due to restricted eye contact

Two therapists reported that diagnostically relevant cues, such as the ability to establish or maintain eye contact, were more difficult to detect in the video setting.

*“How well someone can maintain eye contact is diagnostically interesting, but you hardly get to observe that in videotherapy.” (Interview 1)*

##### 3.2.3.3 Gaze behavior is perceived as challenging

Five therapists reported that they often could not clearly interpret their patients’ gaze direction. It was often unclear whether patients’ attention was focused on the conversation or if they were thinking, looking out of the window or using another program on their screen simultaneously. Furthermore, four psychotherapists perceived the video setting as more distracting, causing both patients and therapists to avert their gaze from the screen and look around the room more frequently.

*“I wondered: Do they perhaps have something else open? Their gaze drifts away like that.” (Interview 11)*

*“I can imagine that this is due to the setting. I have a few patients who look around a lot in their own homes.” (Interview 6)*

##### 3.2.3.4 The video setting is perceived as both confrontational and relieving

One therapist described the gaze directed continuously at the screen in the video setting as uncomfortable because it feels forced and confrontational. She felt that the pauses, silences or natural avoidance of eye contact that usually occur during FtF sessions, such as when discussing emotionally challenging topics, were limited in the digital setting. In contrast, another therapist perceived the opposite, finding the video setting less confrontational and seeing advantages in reduced eye contact for socially insecure patients.

*“In a FtF setting, there is more opportunity to occasionally look away, particularly when discussing difficult topics. On screen, it feels more confrontational and obligatory.” (Interview 7)*

*“Perhaps it is an advantage not to look each other directly in the eyes, particularly for insecure patients.” (Interview 9)*

##### 3.2.3.5 Eye contact and gaze behavior results summary

Comparable to FtF therapy: Two psychotherapists reported no relevant differences in eye contact, provided that camera quality was adequate.

Challenges: Eight psychotherapists described eye contact as challenging and more difficult to establish in specific situations. Two participants reported diagnostic challenges resulting from these limitations. Five psychotherapists stated that the direction of gaze was difficult to interpret, making it unclear whether patients’ attention was focused on the therapeutic interaction. Four psychotherapists also observed more frequent shifts of gaze away from the therapeutic interaction in both themselves and their patients. One psychotherapist described eye contact in the video setting as more forced and confrontational.

Perceived advantages: One psychotherapist perceived eye contact as less forced and less confrontational in the video setting, which was considered beneficial for more insecure patients.

Adaptation strategies: Two psychotherapists reported intentionally looking into the camera rather than at the screen to create a stronger sense of eye contact for their patients.

#### 3.2.4 Body posture

##### 3.2.4.1 Limited interpretive and intervention possibilities regarding body posture

Six therapists reported that the restricted view of the body in the video setting made interpreting body posture more difficult. In particular, the limited camera frame hindered the assessment of tension, nervousness, or restlessness. The initial impression when entering the room and the patient’s posture at the start of therapy were entirely lost. Five therapists reported, that body-oriented therapeutic interventions, such as guided standing or adopting new postures, were difficult to instruct and implement in the video setting. Overall, body posture was perceived as less dynamic and flexible.

*“In person, it’s helpful to move around the room or lie down, but online the possibilities are restricted, the opportunity to use the body as an organ and instrument of expression is limited.” (Interview 5)*

##### 3.2.4.2 Proxemic cues lose their impact in a video setting

Four therapists reported that proxemic cues, such as leaning forward to signal attention, were more challenging to convey. One therapist described leaning toward the camera as feeling unusual and unnatural, while another said they used forward-leaning more for comfort than as a deliberate therapeutic tool. A third therapist explained that she tried to lean forward and backward during sessions to convey non-verbal signals. However, she expressed doubts about how well these movements were perceived by patients and whether they achieved the intended effect.

*“I can’t use the space. If I lean forward on video, it just looks odd. Whether I’m very close or further away, it simply doesn’t have much impact. There’s no real sense of spatial proximity.” (Interview 1)*

##### 3.2.4.3 Changes in body posture in the video setting:

Three therapists reported that they tended to sit more forward-leaning and appear more tense in the video setting. They believed that this could have a negative impact on authenticity and relaxation. Conversely, two therapists observed themselves or their patients adopting a more relaxed and casual posture, such as crossing a leg or sitting more comfortably. They considered this to be a potential risk factor for reduced concentration. One therapist hypothesized that relaxed body posture in a familiar environment could benefit some patients by helping them to open up and feel more comfortable.

*“I sit differently, more forward-leaning. This also changes my posture […]. I feel like I have to be more actively engaged.” (Interview 9)*

*“On video, I sometimes sit cross-legged or with my foot up because I know no one can see me and I find it more comfortable. But it’s not really ideal for the working atmosphere.” (Interview 10)*

##### 3.2.4.4 Body posture results summary

Challenges: Six psychotherapists perceived body posture as more difficult to assess in the VPT, making it harder to accurately interpret signs of tension and nervousness. Five participants reported that body-oriented therapeutic interventions were limited or not feasible in VPT. Four psychotherapists described difficulties in conveying proximity and distance signals, such as leaning forward to express attentiveness. Three participants reported that they tended to sit more leaned forward and more tense during video sessions, which they perceived as potentially reducing authenticity and relaxation. Two psychotherapists noted that the more relaxed posture observed in some patients at home could potentially be associated with reduced attention and concentration.

Perceived advantages: One psychotherapist hypothesized that a relaxed body posture in a familiar environment could benefit some patients by helping them feel more comfortable and open up more easily.

Adaptation strategies: One psychotherapist reported consciously varying their posture by leaning forward and backward during conversations to communicate proximity and distance signals more clearly.

#### 3.2.5 Self-view

##### 3.2.5.1 Adaptation to one’s own video image

Four therapists reported that they initially found their own video image slightly distracting at the beginning of videotherapy. Over time, however, they became accustomed to it. They noted that their own image was usually used only unconsciously as a reassurance, for example, to check the framing. Otherwise, it no longer played a significant role and was generally hardly noticed.

*“At the beginning, the image was distracting; now I only use it briefly to check the screen setup, but otherwise I am fully focused on the patient.” (Interview 3)*

##### 3.2.5.2 Self-viewing can lead to distraction and a loss of therapeutic interaction

Six therapists reported that viewing themselves was distracting, both for themselves and their patients. They noted that their gaze would occasionally drift to their own image. This could interfere with the therapeutic interaction, reducing spontaneity and leading to increased self-monitoring of non-verbal behavior. Some therapists turn off self-viewing at the start of the session and recommend that their patients do the same. Therapists also expressed concern that self-viewing may place an additional burden on patients with low self-esteem or a negative self-image, particularly younger or insecure patients.

*“This contact, which sometimes arises spontaneously in therapy, is distorted when you are constantly observing yourself.” (Interview 11)*

##### 3.2.5.3 Emotional self-regulation through self-observation

Two therapists suggested that self-viewing might lead patients to regulate their emotions more quickly, before the therapist can perceive them. One therapist noted that emotional reactions, such as crying, appeared less frequently and for shorter durations in the video setting.

*“When someone is sad, I usually notice it nonverbally and address it. But when patients see themselves, I can imagine that they regulate their emotions before I even notice anything.” (Interview 11)*

##### 3.2.5.4 Increased self-observation is perceived as beneficial by some therapists

Two therapists reported that they found it helpful to see their own image during videotherapy. It allowed them to monitor their facial expressions and adjust the camera framing if needed, providing a sense of security and professional presence.

*“I don’t think it’s bad to observe myself. It helps me keep track of my own facial expressions and gestures.” (Interview 8)*

##### 3.2.5.5 Self-viewing as a potential therapeutic intervention and diagnostic cue

Three therapists discussed the idea of using self-viewing deliberately as a therapeutic tool. One therapist reported that patients’ reactions to seeing themselves can provide valuable diagnostic insights.

*“Perhaps this is even an advantage for certain patients, for example those with autism, to discuss body language or self-expression without needing a mirror in the room.” (Interview 1)*

*“There are patients who immediately say, “I have to turn off the video; I don’t like seeing myself.” That is also diagnostically very valuable.” (Interview 8)*

##### 3.2.5.6 Self-view results summary

Comparable to FtF therapy: Four psychotherapists reported that they used their own video image only unconsciously for reassurance and otherwise considered it to have little relevance for the therapeutic interaction.

Challenges: Six psychotherapists perceived the self-view as distracting and detrimental to authentic interaction. They expressed concerns that it could place an additional burden on patients with low self-esteem or a negative self-image. Two psychotherapists further suggested that the self-view might promote emotional self-regulation.

Perceived advantages: Two psychotherapists considered the self-view helpful for monitoring their own facial expressions. Three participants suggested that it could also be used deliberately as a therapeutic tool.

Adaptation strategies: Two psychotherapists recommended turning off the self-view during video sessions.

#### 3.2.6 Additional aspects of NVC in the VPT

##### 3.2.6.1 Absence of haptic cues is perceived as a challenge

Three therapists emphasized the complete absence of tactile cues in VPT. For example, it is not possible to shake hands at the beginning of a session, which can also result in the loss of diagnostic value. Two therapists further reported that, especially in emotionally challenging situations, they would like to establish physical closeness, such as placing a hand on the shoulder or, in rare cases, giving a hug. In VPT, this is not possible, leading them to be more cautious when addressing emotionally difficult topics.

*“In trauma therapy, this is even more difficult. I can’t hold anyone online. Especially when someone dissociates […]. I have a patient who needs a firm hug, which I obviously cannot provide online.” (Interview 2)*

##### 3.2.6.2 Frequent misinterpretation of non-verbal cues

Eight therapists mentioned that non-verbal cues were more frequently misinterpreted in VPT. Emotions such as fear or nervousness were often overlooked or misinterpreted; for instance, fidgeting legs or restless chair movements were not visible. Sadness was also reported to be harder to detect. One therapist noted that she only realized her patient was crying when they began to sob audibly. Others reported that tears could generally be more easily overlooked. Assessing patients’ physical condition was also more difficult. One therapist was shocked to see a patient in person after a long time, as they had deteriorated significantly, which had not been noticeable via video. Additionally, two therapists highlighted that the sense of smell is absent in VPT, resulting in the loss of important diagnostic cues regarding hygiene and self-care.

*“There are patients whose appearance suggests poor personal hygiene. You may not notice it immediately, but body odor can sometimes indicate neglect.” (Interview 11)*

##### 3.2.6.3 Increased verbalization of non-verbal cues in the video setting

Six therapists described that they verbalize non-verbal cues such as gestures, facial expressions, or body posture more explicitly. To ensure that their non-verbal signals are understood, they more frequently provide explicit explanations, directly articulate emotional responses such as empathy or interest, and ask more often about patients’ well-being. Patients are also actively encouraged to describe their bodily sensations and experiences verbally to compensate for missing non-verbal information. Therapists further emphasized that it is helpful to jointly reflect on changes in communication behavior in the video setting and to openly discuss potential limitations regarding proximity, visibility, or body language.

*“If I am a little disappointed and think that this disappointment would be noticeable in the room, in videotherapy I would explain it with many words. I want to make sure it comes across to the other person. […] I try to compensate for it with many words.” (Interview 12)*

##### 3.2.6.4 Additional aspects of NVC in VPT results summary

Challenges: Three psychotherapists reported a loss of non-verbal information due to the absence of haptic cues. Eight participants described increased uncertainty when interpreting non-verbal signals and greater concern about misinterpreting them, particularly signs of sadness, tension, and nervousness. Several psychotherapists also reported difficulties in assessing patients’ physical condition and overall appearance.

Adaptation strategies: Six psychotherapists reported verbalizing non-verbal cues, such as gestures, facial expressions, and body posture, more explicitly during video sessions.

##### 3.2.6.5 Recommendations for colleagues

At the end of the interviews, therapists were asked to provide recommendations for their colleagues, particularly regarding the management of NVC in VPT. Therapists who provide both video and in-person therapy emphasized the importance of seeing patients FtF at regular intervals whenever possible. Such meetings facilitate a comprehensive overall impression. It was also frequently recommended to verbalize non-verbal signals more explicitly and to ask clarifying questions when there is uncertainty about emotions. Some therapists suggested deliberately increasing one’s own non-verbal expressivity, for example, by smiling more or nodding more frequently, and observing how this affects patients. They also encouraged being experimental and actively using the specific affordances of the medium, such as positioning the camera to better implement interventions involving body posture or movement.

## 4 Discussion

### 4.1 Principal results and comparison with prior work

This study examined how psychotherapists perceive features of NVC, particularly facial expression, gestures, eye contact, body posture and self-view in the context of VPT. The results suggest that therapists perceive certain aspects of NVC in VPT as restricted and altered. The most frequently reported issues were reduced eye contact, limited visibility of gestures, body posture and increased misinterpretation of non-verbal cues. However, potential advantages were also noted, such as the ability to observe facial expressions more closely or to use one’s own video image deliberately. Thus, the study provides a deeper understanding of how the video setting affects the perception and significance of non-verbal signals. Building on previous research indicating changes in non-verbal processes in VPT (Duffy and Benotsch, 2025), these findings highlight that NVC is not only reduced, but also qualitatively altered. This underlines the importance of considering non-verbal aspects in VPT more systematically and investigating them in greater detail in intervention studies.

Regarding facial expressions, our findings suggest that therapists experienced both limitations and potential advantages in perceiving emotional cues during VPT. In line with observations reported in previous studies, several interviewed therapists perceived subtle emotional cues in facial expressions as more difficult to identify during VPT, particularly due to technical limitations and suboptimal video quality. Similarly, some therapists reported difficulties in recognizing subtle emotional indicators, such as tears or signs of sadness (Duffy and Benotsch, 2025). Conversely, a few therapists noted that, under stable connection conditions, facial expressions could sometimes be analyzed in greater detail than in FtF sessions. They perceived this increased visibility of facial cues as a potential advantage that could partly compensate the reduced visibility of gestures. At the same time, some therapists described that the close-up view of the face could also create a sense of increased intimacy, which they considered potentially challenging for socially insecure or vulnerable patients and the therapeutic relationship. Given these differing perceptions, we suggest that further research should examine how factors such as video quality, camera positioning, and interpersonal distance influence the perception of facial expressions and their role in the therapeutic relationship.

A different pattern emerged for gestures, where therapists predominantly described limitations in the visibility and interpretation of bodily signals during VPT. This is consistent with previous research indicating that restricted visibility of bodily cues may contribute to misinterpretations in VPT. Previous studies have shown that limited observation of bodily signals can result in delayed recognition of emotional reactions or inaccurate assessments of physical states (Ahn and Scheidt, 2023; Hoffmann-Lamplmair et al., 2024). Since the perception of emotional signals influences the therapy process and outcomes (Abargil and Tishby, 2022), we interpret these findings as suggesting that limitations in gesture visibility and potential misinterpretations in the video setting may represent factors that could influence the therapeutic relationship and treatment outcomes negatively. We suggest that it may be beneficial to further explore technical and environmental conditions that enable a more comprehensive view of gestures and body posture. This could, for example, be achieved through wider camera framing or flexible positioning systems.

A central aspect concerns the altered eye contact. Therapists described the asynchronous nature of eye contact in VPT as distracting and unsettling. Similar observations have been reported in studies of other therapy contexts (Duffy and Benotsch, 2025). Previous research suggests that eye contact contributes to the development of trust and plays a key role in empathic communication (MacDonald, 2009). Taken together, we interpret these findings as suggesting that this limitation may represent a potential challenge for the assessment of emotional cues, the diagnostic interpretation of gaze behavior, and the emotional depth of therapeutic interactions. Initial experimental studies provide preliminary evidence that therapists displaying direct eye contact in video-based simulations are perceived more positively by observers (Pfender and Caplan, 2023). Based on previous literature and the findings of our study, we consider that future technological developments, such as adaptive camera positioning or AI-assisted gaze alignment, could be worthwhile areas for further investigation to explore whether they may facilitate a more natural experience of eye contact and support trust and empathy in VPT.

Another central finding concerns changes in non-verbal communication due to self-viewing. Overall, most of the therapists perceived NVC as less authentic and more controlled when patients could see themselves. Some therapists expressed concerns that this effect may be particularly challenging for patients who already experience low self-esteem or a negative self-image, as increased self-focus could potentially intensify self-consciousness and make emotional openness more difficult. Some therapists also hypothesized that self-viewing may lead to early self-regulation of emotional responses, potentially reducing spontaneous emotional expressivity. Previous research suggests that patients’ emotional expression during psychotherapy is positively associated with treatment outcomes (Peluso and Freund, 2018). Building on this evidence and the therapists’ reflections in our study, we speculate that self-viewing may reduce spontaneous emotional expression and could therefore represent a potential mechanism through which the treatment outcome may be influenced. Other therapists described also advantages, particularly the possibility of using the own video image as a tool to increase awareness and support the development of non-verbal skills. We think that this perspective may be particularly relevant for specific patient groups, such as individuals with autism spectrum disorders, for whom structured support in recognizing and regulating emotional and social signals may be beneficial. Recent telehealth-based interventions in autistic children and adolescents have highlighted the potential of digital approaches to support emotion regulation skills (Coffman et al., 2024). The present findings indicate that self-viewing may have a complex role in VPT, as some therapists perceived potential benefits, while others raised concerns regarding increased self-focus and possible effects on emotional expressivity. In our view, self-viewing appears to play a complex role in VPT. We therefore suggest that it may be worthwhile to explore for which patient groups self-viewing might be beneficial and under which circumstances disabling it may be preferable.

Some therapists deliberately adapted their NVC, for example by smiling more frequently or nodding more emphatically. Many also reported a conscious increase in verbal expressions, used as a compensatory strategy for the loss of non-verbal cues, intended to convey emotional content and therapeutic messages more clearly. Evidence on the deliberate enhancement of non-verbal expressivity or increased verbalization remains limited. Initial findings, however, suggest that greater non-verbal expressiveness is positively associated with therapy outcomes (Peluso and Freund, 2018). Therapists with higher non-verbal emotional expressivity also rated their competencies in videotherapy as less restricted compared with colleagues with lower expressivity (Henry et al., 2012). In our interpretation, it is conceivable that greater non-verbal expressivity may not only enhance therapists’ perception of their own abilities but also encourage patients to express their own non-verbal cues and associated emotional responses more clearly. We further speculate that this process could partially compensate for the self-monitoring induced by self-viewing and, in turn, promote emotional resonance in the therapeutic exchange.

### 4.2 Limitations

The inclusion criteria for this study were designed to recruit experienced therapists primarily, in order to gain a realistic and detailed understanding of their experiences with VPT. Consequently, psychotherapists with comparatively little experience of VPT were not included. Nevertheless, the sample covered a relatively wide range of experience, from a few years to over 30 years. The findings are based on a relatively small sample of 12 psychotherapists and should therefore be interpreted with appropriate caution. In addition, the study was conducted in Germany and included therapists working within the German healthcare system in public healthcare and private practice settings. As psychotherapeutic practice, healthcare systems, and communication norms may differ across countries and cultural contexts, the transferability of the findings to other settings may be limited. Only around 10% of contacted therapists responded, half of whom were unable to participate due to time constraints. This may have resulted in an overrepresentation of therapists with a particular interest in videotherapy, while those with more skeptical views may have been underrepresented. As a result, certain aspects of non-verbal communication in VPT may have been perceived and described as less problematic or more favorable than they might have been in a more diverse sample, which may limit the transferability of the findings. Due to the low response rate, not all therapeutic approaches could be included. However, three of the four most commonly practiced approaches in Germany, cognitive behavioral therapy, psychodynamically oriented psychotherapy and psychoanalysis, were represented, ensuring a broad overview. Although regional, age-related and generational factors were not fully representative, the sample included therapists from diverse regions across Germany and covered a wide age range (29–61 years). As the interviews were conducted via video, comparable to the VPT sessions themselves, the medium may have influenced participants’ responses. For instance, participants may have paid more attention to their own and the interviewer’s NVC, resulting in more detailed observations in this regard. Finally, although AI-assisted transcription and generative AI tools were used to support transcription, language editing and translation, all transcripts, quotations, and translations were manually reviewed by the authors against the original interview material to ensure that the participants’ intended meaning was accurately preserved. This procedure was intended to support the trustworthiness and credibility of the qualitative analysis.

### 4.3 Clinical and research implications

Due to the differences reported regarding video and FtF psychotherapy in NVC, we suggest that clinicians remain mindful of the changes inherent in the video setting and consider explicitly addressing potential differences in non-verbal cues with patients in a psychoeducational manner. For example, information typically conveyed through hand movements may be less visible, and actively observing or inquiring about such cues could support understanding. Drawing on the therapists’ experiences reported in our study, we encourage exploring the deliberate use of non-verbal signals, such as increased smiling or nodding, to gain insight into how these behaviors may shape patients’ perceptions and engagement.

Based on the findings of our study, we suggest that future research should further explore the perceived limitations and challenges of NVC in videopsychotherapy, ideally using quantitative methods to complement qualitative insights. Our results indicate that it may be valuable to examine whether and how these challenges relate to the therapeutic relationship and treatment outcomes. In addition, therapists’ reflections on self-viewing suggest that this represents another important area for future research. Future studies could explore how self-viewing may influence the therapy process and under which conditions activating or deactivating self-viewing might be therapeutically beneficial. Camera positioning also warrants consideration, as an appropriate distance may allow clear visibility of gestures and facial expressions while avoiding a sense of over-intimacy. Furthermore, the identified potentials, such as the deliberate use of non-verbal signals or intervention-specific adaptations for particular clinical populations, should be tested and evaluated in real-world therapy settings. It may also be valuable to examine how deliberately enhancing non-verbal expressivity affects patients, the therapeutic relationship, and treatment outcomes. The present study focused on a limited number of aspects of non-verbal communication. Nevertheless, we consider it conceivable that additional non-verbal and paraverbal features, such as silences, tone of voice, and vocal clarity, may be relevant for therapeutic interactions and could therefore be valuable areas of investigation in future research on videopsychotherapy.

## 5 Conclusion

This study extends previous research by providing an in-depth exploration of psychotherapists’ perceptions of specific aspects of NVC in VPT, including eye contact, facial expressions, gestures, body posture, and self-view. The findings indicate that these aspects are perceived as being affected by VPT to varying degrees. Some aspects, such as eye contact and gestures, were frequently described as restricted due to the characteristics of the video format, which may increase the risk of misunderstandings and negatively affect the therapeutic process. Other aspects of NVC were perceived by some therapists as largely comparable to those in FtF psychotherapy. In addition, certain aspects, particularly patients’ facial expressions, were perceived by some therapists as easier to observe in VPT. In response to these changes, therapists reported adapting their communication and therapeutic practice by using their own verbal and NVC more deliberately and explicitly.

We therefore recommend discussing possible differences in NVC in VPT transparently with patients as part of psychoeducation. To better support altered NVC in VPT, future research could investigate how camera settings, self-view options, and training in non-verbal expressivity influence the therapeutic relationship and treatment outcomes. Such studies could help clarify whether and how NVC should be given greater consideration in guidelines and training for VPT.

## Data Availability

The original audio recordings are not available in order to protect participants’ privacy and confidentiality. Anonymized interview transcripts may be made available by the corresponding author upon reasonable request, subject to applicable ethical and data protection requirements.
