# Supplementary material for: Therapists’ experiences with non-verbal communication in videopsychotherapy: a qualitative interview study
Source: Front Psychol. 2026 Aug 24;17:1785382. doi: 10.3389/fpsyg.2026.1785382 (PMC13547077; doi:10.3389/fpsyg.2026.1785382)
Supplement: Supplementary file 1 [file Supplementary_file_1.docx]

| Definition of Nonverbal Communication: The term nonverbal communication encompasses all signals that are emitted by a person outside of the spoken content and can be perceived and interpreted by another person (1). Since the entirety of nonverbal signals would exceed the scope of this research project, our interviews will focus primarily on the nonverbal signals that can be observed through the eyes, specifically facial expressions, gestures, and eye behavior. However, if other nonverbal signals occur to you during the course of our conversation that you consider important, please feel free to share them with me.  **Supplementary Material 1: Semi-structured interview guide** | | | |
| --- | --- | --- | --- |
| Interview Questions | **Checklist –** Was it mentioned? Memo for possible follow-up questions – only ask if the points were not spontaneously mentioned | **Specific questions** – ask at the appropriate point in this formulation | **Maintenance and tax questions** |
| 1. At the beginning of the interview, I would like to ask you about your attitude towards video-based psychotherapy. | - Attitude towards video psychotherapy - Frequency of use | - **In what context are you currently using video psychotherapy?** | - Could you tell me more about your experiences? - Is there anything else? - What are your reasons for treating patients via video? - Under what conditions do you take patients into the online setting? |
| I would now like to talk to you about nonverbal communication in video psychotherapy.   2. How relevant is nonverbal communication when interacting with your patients? | - Relevance of nonverbal communication in direct contact - Relevance of nonverbal communication via video | ·  **How relevant is nonverbal communication when communicating with your patients via video?**  -> If relevance varies: Why would you attribute greater/lesser relevance to nonverbal communication in an online setting? | - Could you explain in more detail why you attribute a rather high importance / a rather low importance to nonverbal communication? |
| 3. What differences and similarities in nonverbal communication do you perceive between face-to-face settings and video settings? | - Differences - Similarities | - **How do differences in nonverbal communication affect your therapy**? | - Is there any other difference that you notice? |
| 4. What advantages and disadvantages do you see in relation to nonverbal communication in a video setting? | - Advantages - Disadvantages | - **Which aspects of nonverbal communication are facilitated by the video setting?** | - What is particularly important for the therapy? - What do you particularly miss in the online setting? |
| I would like to talk to you in more detail about three specific aspects of non-verbal communication in a video setting.   5. What do you think is special about eye contact, facial expressions and gestures in a video setting? | - Eye contact - Facial expression - gestures | - **Which aspect of nonverbal communication do you consider particularly important in an online setting, and why?** | - Is there anything else that strikes you as unusual about video? - Can you think of any other aspects? |
| 6. How do you perceive your patients' nonverbal communication in a video setting? | - Interpretation of nonverbal signals | - **Are there any problems or challenges in perceiving the nonverbal signals your patients send out?** - *-> If so, which signals or situations does this particularly affect?* - **Are there any non-verbal signals that your patients use more or less frequently?** | - You mentioned... Could you give us an example? - Have there ever been any misunderstandings in the online setting? - Is there another aspect? |
| 7. How do you perceive your own nonverbal communication in a video setting? | - Own nonverbal communication - Specifics of one's own nonverbal communication | - **Do your patients have problems understanding or interpreting the nonverbal signals you send as a therapist?**   *-> If so, which situations or signals does this particularly affect?*   - **To what extent do you consciously use aspects of nonverbal communication in an online setting?** - **How does being able to see your own image during a video conference affect you?** | - Can you think of any other changes? - Have any of your patients experienced misunderstandings in the online setting? - Can you give an example of a specific situation? |
| 8. How do you experience the influence of nonverbal communication on the therapeutic relationship in a video setting? | - Impact on the therapeutic relationship - Differences from face-to-face settings |  | - Can you describe this in more detail? - Can you think of a situation that illustrates this? |
| 9. Imagine that a less experienced colleague asks you for tips for their first online therapy sessions. What advice would you give them? | - General recommendations / tips | - **Are there any recommendations you would give for optimising nonverbal communication in an online setting?** | - Are there any other points you would change? - Are there any points you would like to see added? |

**Is there anything else you would like to add on the subject of video-based psychotherapy and nonverbal communication?**

**Demographic data:**

To conclude the interview, I would just like to ask you a few more questions, and then we will be done.

1. How old are you? …………………………………………………………………
2. Which gender do you identify with?
   - male
   - female
   - divers
3. Which professional qualification are you aiming for, or which qualification have you already completed?
- Cognitive Behavioral Therapy
- Psychodynamic Psychotherapy
- systemic psychotherapy
- Psychoanalysis
  4. When did you complete your training or in which year do you expect to complete your training?
- Psychotherapeutic licensure training already completed in the year:: ………………………………………………
- Expected completion in:: …………………………….
  5. How many years have you been working as a psychotherapist?
- Less than 5 years
- More than 5 years
- More than 10 years
  6. In which area are you currently working (multiple options possible)
- Inpatient
- Outpatient
- Private practice
- Other: ………………………………………………………..
  7. In which federal state do you currently practise? ……………………………………………………………………………
  8. How many different patients have you treated via video so far?
- Less than 5 patients
- Less than 10 patients
- More than 10 patients
- More than 20 patients
- More than 50 patients
  9. How many therapy sessions have you completed via video so far?
- More than 10 therapy sessions
- More than 20 therapy sessions
- More than 30 therapy sessions
- More than 40 therapy sessions
- More than 50 therapy sessions
  10. Which disorders do you treat using video psychotherapy? …………………………………………………………………………………………………………………………………………………………………………………………………………
  11. What proportion of all your therapy sessions is video psychotherapy?
- Less than 10%
- More than 10%
- More than 20%

References:

1.	Argyle M. Körpersprache & Kommunikation: nonverbaler Ausdruck und soziale Interaktion. 10., überarbeitete Neuauflage. Paderborn: Junfermann; 2013. 1 p.
