# Supplementary material for: Therapists’ experiences with non-verbal communication in videopsychotherapy: a qualitative interview study
Source: Front Psychol. 2026 Aug 24;17:1785382. doi: 10.3389/fpsyg.2026.1785382 (PMC13547077; doi:10.3389/fpsyg.2026.1785382)
Supplement: Supplementary file 2 [file Supplementary_file_2.docx]

| Category & Subcategory  **Supplementary Material 2: Code System** | Definition | Example |
| --- | --- | --- |
| Attitude toward video psychotherapy | Direct statements about the interviewees' opinions, attitudes, and beliefs regarding video psychotherapy  Both positive and negative assessments and personal preferences | "For me, video therapy is very useful and I find it very effective overall, so I would say that I have a positive attitude towards it." |
| Usage behavior | All statements on the use of video therapy in the past and present, as well as the course of usage behavior | "I currently only use video therapy because it is much more time-efficient and location-independent for me." |
| Verbalization | All statements about verbalization in video therapy, including increased or decreased verbal interaction between therapists and patients, content-related aspects, and changes in topics addressed in the online setting. | "Since I can't see the patients very well, I ask more questions in the video setting about what their feet are doing or how they are sitting on the chair." |
| Positive aspects of video therapy | All positive aspects of video therapy, advantages, opportunities, possibilities, and potential enabled by video therapy and not related to nonverbal communication. These include, for example, good accessibility, independence... | "Video therapy enables me to treat patients from abroad and allows more patients to benefit from therapy." |
| Negative aspects of video therapy | All negative aspects, challenges, or problems in video therapy that are made possible by video therapy and are not related to nonverbal communication. These include, for example, technical aspects, challenges due to distractions such as noise, reduced concentration and attention, etc. | "Technical aspects are often difficult to implement, the connection frequently breaks down, and therapy online is more difficult with people who have no technical experience." |
| Setting & space: differences | This category includes all statements relating to differences between video and face-to-face therapy, without any value judgment (no distinction between positive or negative). For example, differences in the atmosphere, the course of therapy, or other framework conditions. | "For some patients, routines from home come into play, such as always making tea before therapy, while others can go straight from work to therapy." |
| Relevance of nonverbal communication in general | General statements about the relevance, importance, and significance of nonverbal communication in therapy that cannot be classified into either of the two subcategories | "Basically, nonverbal communication is highly relevant in therapy, as patients always communicate through it, consciously and unconsciously. I don't see any difference between online and face-to-face therapy in this respect." |
| - *Relevance of nonverbal communication in face-to-face therapy* | All direct statements about the relevance of nonverbal communication in face-to-face therapy (in-person therapy).  All statements and comparisons that imply that nonverbal communication is more relevant/important in face-to-face therapy than in video therapy. | "Nonverbal communication is not quite as important in video therapy as it is in face-to-face therapy, as you see less of the body. In contrast, I perceive the patient much more strongly in face-to-face therapy with their overall appearance, posture, and gestures." |
| - *Relevance of nonverbal communication in video therapy* | All direct statements about the relevance of nonverbal communication in video therapy.  All statements about comparisons that imply that nonverbal communication is more relevant in a video setting than in a face-to-face setting. | "In face-to-face therapy, I don't find nonverbal communication as important, because you have many more signals than in online therapy, where you have to focus more on the few nonverbal signals that are there." |
| Eye behavior and eye contact | To be understood as an umbrella category for statements about the perception, meaning, and interpretation of eye contact and gaze direction in therapy that cannot be classified in the subcategory "positive or negative aspects."  Similarities in eye behavior or eye contact that do not allow for a positive or negative weighting. | "In terms of eye contact, I did not notice any difference between the two settings." |
| - *Positive aspects of eye behavior and eye contact in the video setting* | All statements about eye behavior and eye contact that, compared to the face-to-face setting, imply that the signals in the video setting are perceived more positively and better or are easier to implement.  Also opportunities and possibilities that are made possible in terms of eye contact/gaze behavior in the video setting. | "Eye contact plays a special role in the video setting, as it is easier for patients to look at my image." |
| - *Negative aspects of eye behavior and eye contact in a video setting* | All statements about eye behavior and eye contact that, compared to face-to-face settings, imply that signals are perceived more negatively or poorly in video settings, are more difficult to implement, or are more likely to lead to problems and challenges. | "Eye contact in a video setting is virtually impossible, so some connection is lost." |
| Facial expressions | To be understood as an umbrella category for statements about the perception, meaning, and interpretation of facial expressions in therapy that cannot be classified in the subcategory "positive or negative aspects."  Statements about similarities or differences in facial expressions that do not allow for a positive or negative weighting.  Also, special features in facial expressions, such as conscious use or other abnormalities. | "I haven't noticed any difference in facial expressions compared to face-to-face settings and can actually recognize everything just as well as in person." |
| - *Positive aspects of facial expressions in the video setting* | All statements about the facial expressions/micro-changes of the patients or therapists themselves which, compared to the face-to-face setting, imply that the signals in the video setting are perceived more positively and better or are easier to implement.  Also opportunities and possibilities that are made possible with regard to facial expressions. | "I do feel that patients find it easier to express their emotions through facial expressions in a video setting than in a face-to-face setting." |
| - *Negative aspects of facial expressions in a video setting* | All statements about facial expressions or micro-expressions that imply that, compared to face-to-face settings, signals are perceived as more negative or worse in video settings, are more difficult to implement, or are more likely to lead to problems and challenges. | "Particularly subtle differences in facial expressions are perceived very poorly or sometimes not at all in a video setting." |
| Gestures | To be understood as an umbrella category for statements about the perception, meaning, and interpretation of gestures in therapy that cannot be classified in the subcategory "positive or negative aspects." Gestures here refer to movements of individual body parts, such as shrugging shoulders, foot, arm, or hand movements that take place below the head.  Statements about similarities, differences, or peculiarities in gestures that do not allow for a positive or negative weighting. | "I didn't really notice any difference in gestures." |
| - *Positive aspects of gestures in the video setting* | All statements about the gestures of the patients or therapists themselves which, in comparison to the face-to-face setting, imply that the signals in the video setting are perceived more positively and better or are easier to implement.  Also opportunities and possibilities that are made possible with regard to gestures. | "I think it's a little easier for patients to gesticulate when speaking." |
| - *Negative aspects of gestures in a video setting* | All statements about gestures that, compared to face-to-face settings, imply that signals in video settings are perceived as more negative or worse, are more difficult to implement, or are more likely to lead to problems and challenges. | "I usually don't notice fidgeting under the table, so a lot of information is lost." |
| *Body posture* | This category includes all aspects of the posture of patients and therapists in a video setting. Posture refers, for example, to the way of sitting, leaning forward and backward, and posture in the room.  It also includes comparisons with posture in face-to-face settings, which do not allow for "positive" or "negative" evaluations. | "I notice that my patients don't sit quite as upright as they do in a face-to-face setting. However, I can't say whether this is relevant." |
| *- Positive aspects of posture in a video setting* | This category includes all aspects of patient and therapist posture in a video setting that allow for a positive assessment.  Also opportunities, potential, and possibilities | "Patients usually sit a little more relaxed than they do in my office, which creates a calmer atmosphere." |
| *- Negative aspects of posture in a video setting* | This category includes all aspects of patient and therapist posture in a video setting that allow for a negative assessment.  Also challenges, difficulties, and risks. | "Patients tend to sit in a more relaxed manner, but I feel that this makes them less focused on the therapy." |
| Further aspects of nonverbal communication | This is to be understood as an umbrella category that includes everything related to nonverbal communication that cannot be classified in one of the other categories of facial expressions, gestures, eye behavior, or posture and does not allow for a positive or negative assessment.  For example, statements about energies in therapy or very general comparisons of nonverbal communication. Also aspects of touch communication such as hugs or handshakes. | "Surprisingly, I can perceive the energy and charisma of the other person just as well via video as I can in face-to-face therapy." |
| *- Misinterpretations* | This category includes all statements about direct misinterpretations of nonverbal signals in video therapy or statements about misinterpretations in comparison to face-to-face therapy. | "Sadness is a feeling that I have probably misinterpreted before, as I often only noticed it later when someone started crying or had red eyes." |
| *- Other positive aspects of nonverbal communication in a video setting* | This mainly includes positive statements, opportunities, and potential in relation to nonverbal communication in video therapy that cannot be assigned to any of the other clear categories such as facial expressions, gestures, posture, or eye behavior. It also includes positive aspects of touch communication such as hugs or handshakes in a video setting. | "Sometimes I feel that I perceive patients' emotions better than in a face-to-face setting, but I can't really explain why that is." |
| *- Other negative aspects of nonverbal communication in a video setting* | These include, above all, negative statements as well as challenges and risks related to other aspects of nonverbal communication that cannot be assigned to any of the other clear categories such as facial expressions, gestures, posture, or eye behavior. This also includes negative aspects of physical communication such as hugging or shaking hands in a video setting. | "Above all, handshakes at the beginning of therapy or hugs during trauma therapy are no longer possible, which I find to be a major limitation." |
| Own video | This is to be understood as an umbrella category in which comments on the perception and influence of one's own video image during online therapy sessions are classified by both therapists and patients, without allowing for a "positive" or "negative" evaluation. | "One difference in video therapy is above all the video image, which is like a mirror throughout, which can be both positive and negative for some patients." |
| - Positive aspects of one's own video | Comments on the perception and influence of one's own video image during online therapy sessions, both from therapists and patients, which allow for a positive evaluation or describe opportunities, possibilities, or potential. | "When patients look at their own video a lot, I think it's a good opportunity to use this therapeutically, as it allows some topics to be addressed more quickly than others." |
| - Negative aspects of one's own video | Comments on the perception and influence of one's own video image during online therapy sessions, both from therapists and patients, which allow for a negative assessment or describe problems, challenges, and risks. | "I notice, especially with younger patients, that their own video sometimes distracts them from the actual therapy." |
| Therapeutic relationship in a video setting | Quality and development of the relationship between therapist and patient in video therapy  For example, emotional openness, trust, working alliance, etc.  Factors that weaken or promote the relationship  Comparisons to in-person therapy | "I feel that patients find it easier to open up in a video setting." |
| - *Influence of nonverbal communication on the therapeutic relationship in a video setting* | All statements about the influence or significance of nonverbal signals in online therapy that relate to the therapeutic relationship. | "In video therapy, I place a lot of importance on showing my smile more clearly. This creates validation and positive reinforcement for the other person in this somewhat rigid setting, which makes patients feel more comfortable and perhaps more likely to confide their feelings to me." |
| Recommendations for action / tips | Specific suggestions and strategies for improving video therapy in general.  Direct tips for other psychotherapists, both general tips for using video therapy and specific tips for optimizing nonverbal communication.  Also, wishes or general suggestions for improvement for the future use of video therapy. | "I can only advise everyone to try as much as possible and get creative; most things work surprisingly well in video therapy." |
