# Supplementary material for: Therapists’ experiences with non-verbal communication in videopsychotherapy: a qualitative interview study
Source: Front Psychol. 2026 Aug 24;17:1785382. doi: 10.3389/fpsyg.2026.1785382 (PMC13547077; doi:10.3389/fpsyg.2026.1785382)
Supplement: Supplementary file 3 [file Supplementary_file_3.docx]

**Supplementary Material 3: COREQ checklist**

Consolidated criteria for reporting qualitative studies (COREQ): 32-item checklist

Developed from:

Tong A, Sainsbury P, Craig J. Consolidated criteria for reporting qualitative research (COREQ): a 32-item checklist for interviews and focus groups. International Journal for Quality in Health Care. 2007. Volume 19, Number 6: pp. 349 – 357

| **Item No** | | **Guide Questions/Description** | **Answer - Reported on Page** |  |  |
| --- | --- | --- | --- | --- | --- |
| **Domain 1: Research team and reflexivity** | | | |  |  |
| **Personal Characteristics** | | | |  |  |
| 1. Interviewer/ facilitator | | Which author/s conducted the interview or focus group? | German speaking medical student. |  |  |
| 2. Credentials | | What were the researcher’s credentials? E.g., PhD, MD | The interviewer was a medical student, the research team included psychotherapists, medical reasearchers, psychologists, qualitative research experts. |  |  |
| 3. Occupation | | What was their occupation at the time of the study? | The interviewer was a medical student at the time of data collection. The research team consisted of clinicians and researchers from the Department of Psychosomatic Medicine, who were engaged in both clinical practice and academic research. |  |  |
| 4. Gender | | Was the researcher male or female? | The interviewer was female, the research team was female and male. |  |  |
| 5. Experience and training | | What experience or training did the researcher have? | The interviewer had basic knowledge of qualitative methods and interview techniques. She had no prior practical experience in videotherapy. |  |  |
| **Relationship with participants** | | | |  |  |
| 6. Relationship established | | Was a relationship established prior to study commencement? | Most therapists had no prior acquaintance with the interviewer. One therapist from the study’s clinic was known by sight only; some therapists were recruited via project collaborators, but no prior personal contact existed to the interviewer. |  |  |
| 7. Participant knowledge of the interviewer | | What did the participants know about the researcher? e.g. personal goals, reasons for doing the research? | Participants were informed that the interviewer was a medical doctoral candidate conducting the study within the “PsyPan” project, with a research focus on nonverbal communication in video-based psychotherapy. |  |  |
| 8. Interviewer characteristics | | What characteristics were reported about the interviewer/facilitator? e.g. Bias, assumptions, reasons and interests in the research topic | The interviewer had no prior experience in video-based psychotherapy, no expected bias, and conducted her first interview study. Interest in the topic stemmed from her doctoral research and general interest in psychotherapy. |  |  |
|  | **Domain 2: study design** | | |  |  |
|  | **Theoretical framework** | | |  |  |
|  | 9. Methodological orientation and Theory | What methodological orientation was stated to underpin the study? e.g. grounded theory, discourse analysis, ethnography, phenomenology, content analysis | The data were analyzed using qualitative content analysis based on Mayring (1). |  |  |
|  | **Participant selection** | | |  |  |
|  | 10. Sampling | How were participants selected? e.g., purposive, convenience, consecutive, snowball | Participants were selected using purposive and snowball sampling. Therapists were identified via internal clinic-wide email, online research, and referrals from other participants. |  |  |
|  | 11. Method of approach | How were participants approached? e.g., face-to-face, telephone, mail, email | Participants were approached via email. An internal clinic-wide email was sent, individually contacted therapists were emailed, and some participants were asked to forward the study information to others. |  |  |
|  | 12. Sample size | How many participants were in the study? | 12 psychotherapists were interviewed |  |  |
|  | 13. Non-participation Setting | How many people refused to participate or dropped out? Reasons? | Over 200 therapists were contacted. The majority did not respond or declined to participate due to time constraints or lack of experience. About 10% showed interest, but some were excluded or unavailable. The exact number of refusals was not recorded. |  |  |
|  | 14. Setting of data collection | Where was the data collected? e.g., home, clinic, workplace | Interviews were conducted online via Zoom. |  |  |
|  | 15. Presence of nonparticipants | Was anyone else present besides the participants and researchers? | Only the interviewer and participant were present during each interview. |  |  |
|  | 16. Description of sample | What are the important characteristics of the sample? e.g. demographic data, date | Participants were psychotherapists with at least one year of professional experience and at least five video-based individual therapy sessions with a minimum of three different patients. The age range was 29 to 61 years. |  |  |
|  | **Data collection** | | |  | No |
|  | 17. Interview guide | Were questions, prompts, and guides provided by the authors? Was it pilot tested? | A semi-structured interview guide based on Helfferich’s model (2) was developed and piloted. It was revised based on expert feedback. |  |  |
|  | 18. Repeat interviews | Were repeat interviews carried out? If yes, how many? | No repeat interviews were conducted. |  |  |
|  | 19. Audio/visual recording | Did the research use audio or visual recording to collect the data? | Interviews were audio-recorded with participant consent. |  |  |
|  | 20. Field notes | Were field notes made during and/or after the interview or focus group? | After each interview, the interviewer completed a structured protocol sheet documenting the interview duration, type of initial contact, interview atmosphere, and any notable occurrences during the session. |  |  |
|  | 21. Duration | What was the duration of the interviews or focus group? | Interviews lasted between 16 and 59 minutes (average: 29 minutes). |  |  |
|  | 22. Data saturation | Was data saturation discussed? | As inductive coding progressed, no further relevant categories emerged, indicating thematic saturation. |  |  |
|  | 23. Transcripts returned | Were transcripts returned to participants for comment and/or correction? | Transcripts were not returned to participants for correction or feedback. |  |  |
|  | **Domain 3: analysis and findings** | | |  |  |
|  | **Data analysis** | | |  |  |
|  | 24. Number of data coders | How many data coders coded the data? | Coding was performed by at least one researcher. A second coder evaluated one transcript for interrater reliability. |  |  |
|  | 25. Description of the coding tree | Did the authors provide a description of the coding tree? | Deductive and inductive categories were developed based on the literature and interview guide; categories were discussed and refined within the team. |  |  |
|  | 26. Derivation of themes | Were themes identified in advance or derived from the data? | Themes were derived both deductively from existing theory and inductively from the data. |  |  |
|  | 27. Software | What software, if applicable, was used to manage the data? | MAXQDA was used for analysis. |  |  |
|  | 28. Participant checking | Did participants provide feedback on the findings? | Participants did not review the findings. |  |  |
|  | **Reporting** | | |  |  |
|  | 29. Quotations presented | Were participant quotations presented to illustrate the themes/findings? Was each quotation identified? e.g., participant number | We aimed to present participant quotes for the most frequently mentioned or most important themes. Some themes were summarized without direct quotes. |  |  |
|  | 30. Data and findings consistent | Was there consistency between the data presented and the findings? | Participant quotes and examples were presented to illustrate the main themes. Each quote was referenced from the corresponding interview transcript. Some minor themes were summarized without direct quotes for clarity, but overall the data presented align with the conclusions drawn. |  |  |
|  | 31. Clarity of major themes | Were major themes clearly presented in the findings? | Yes, the major themes were clearly presented. We structured the findings with headings, starting with the most frequently mentioned and important points, followed by less frequently mentioned points within each theme. |  |  |
|  | 32. Clarity of minor themes | Is there a description of diverse cases or a discussion of minor themes? | Yes, less frequently mentioned aspects were also addressed and discussed |  |  |

References:

1.	Mayring P, Fenzl T. Qualitative Inhaltsanalyse. In: Baur N, Blasius J, editors. Handbuch Methoden der empirischen Sozialforschung [Internet]. Wiesbaden: Springer Fachmedien Wiesbaden; 2019 [cited 2024 Feb 29]. p. 633–48. Available from: http://link.springer.com/10.1007/978-3-658-21308-4_42

2.	Helfferich C. Die Qualität qualitativer Daten: Manual für die Durchführung qualitativer Interviews. 4. Auflage. Wiesbaden: VS, Verl. für Sozialwiss; 2011. 214 p. (Lehrbuch).
